# A New Experimental Device for Transapical Access of the Aortic and Mitral Valves as well as the Aorta in its Various Segments

**DOI:** 10.21470/1678-9741-2017-0013

**Published:** 2017

**Authors:** Leonardo Paim, José Honório Palma da Fonseca, Francismar Vidal de Arruda Junior, Paulo Sampaio Gutierrez, Luiz Felipe Pinho Moreira, Fabio Biscegli Jatene

**Affiliations:** 1 Instituto do Coração do Hospital das Clínicas da Faculdade de Medicina da Universidade de São Paulo (InCor-HCFMUSP), São Paulo, SP, Brazil.

**Keywords:** Models, Animal, Hemorragia, Endovascular Procedures, Stents, Minimally Invasive Surgical Procedures

## Abstract

**Objective:**

To present the results of a new experimental device developed to facilitate the transapical access in endovascular treatment of structural heart diseases. It aims to reduce the risk of bleeding and complications in this type of access and demonstrate the device as a safe, fast and effective alternative.

**Methods:**

CorPoint is composed of three parts: introducer, base with coiled spring, and closing capsule. By rotating movements, the spring is introduced into the myocardium and progressively approaches the base to the surface of the heart. Guidewires and catheters are inserted through the hollow central part and, at the end of the procedure, the capsule is screwed over the base, therefore stopping any bleeding.

**Results:**

The device was implanted in 15 pigs, weighing 60 kg each, through an anterolateral thoracotomy, while catheters were introduced and guided by fluoroscopy. All animals had minimal bleeding; introducers with diameter up to 22 Fr were used and various catheters and guidewires were easily handled. After finishing the procedure, the closing capsule was attached and no bleeding was observed at the site.

**Conclusion:**

This new device has proved effective, fast and secure for the transapical access. This shows great potential for use, especially by ensuring an easier and direct access to the mitral and aortic valves; the shortest distance to be traveled by catheters; access to the ascending and descending aorta; decreased bleeding complications; decreased surgical time; and the possibility of allowing the technique to evolve and become totally percutaneous.

**Table:** 

Abbreviations, acronyms & symbols	
TAAVI	= Transapical transcatheter aortic valve implantation
TAVI	= Transcatheter aortic valve implantation
TFAVI	= Transfemoral transcatheter aortic valve implantation

## INTRODUCTION

The treatment of structural cardiovascular diseases, such as aortic stenosis, mitral valve degeneration, aortic aneurysms and dissections, as well as mechanical circulatory assistant devices have been performed through minimally invasive procedures in an ever so growing manner. Since the first case of transcatheter aortic valve implantation (TAVI) performed by Doctor Allan Cribier, in 2002^[1]^, the number of patients who receive catheterbased heart valves and aortic endografts has been growing exponentially.

Regarding structural heart diseases, transcatheter aortic valve implantation is undisputedly the main example of this type of approach. With more than 100.000 procedures worldwide, it has been established as the main option for the treatment of symptomatic aortic stenosis in inoperable and high risk patients^[2-5]^, with newer studies trying to push this indication towards moderate risk patients, such as the PARTNER 2 Trial^[6]^.

TAVI can be performed through various access routes, and while transfemoral approach is the most common, other options are widely available and performed according to each center's expertise, such as transaxillary/subclavian, transaortic, transcarotid, and the main option, transapical approach^[7,8]^. Although both transfemoral and transapical techniques are well established as viable access routes for TAVI and other endovascular procedures such as valve-in-valve implants, each one presents its difficulties, peculiarities and complications, making it difficult so far to establish the superiority of one over the other, when both options are feasible^[9]^.

The transfemoral approach is considered to be less invasive, performed through a puncture of the common femoral artery, ideally at its bifurcation point and, due to recently available assistant closure devices, this technique can be performed in a totally percutaneous manner. On the other hand, the transapical approach guarantees an easier access to the aortic and mitral valves, as well as the ascending aorta, in a way that the catheters are less introduced and have to travel a much smaller distance, which makes it easier to manipulate and position them. However, this approach requires a thoracotomy, general anesthesia and the placement of sutures at the apex of the left ventricle, thus characterizing a more invasive procedure. Furthermore, serious hemorrhage can occur due to the manipulation of the left ventricle, be it during the procedure or postoperatively, which accounts for one of the main concerns regarding this method.

Other advantages of the transapical approach are cited below^[3]^:

1. Presents no vascular caliber limitations and can be performed in patients with inaccessible aorto-iliac systems, either as a consequence of severe calcification or aneurysmal dilatations.
2. Smaller risk of micro-embolization by less trauma to the stenotic aortic valve, when compared to the retrograde course.
3. Allows implant of the valve with no previous dilatation.
4. Utilizes wider catheters, which translates in lesser need to crimp the valve and therefore, less trauma to the prosthesis. This may increase its durability throughout time.
5. The position of the prosthetic valve over the native valve is better defined and easier to be implanted, since the angle of the catheter's insertion is much more favorable.
6. In re-operations, the adhesion that is created between the left ventricle and the pericardium, in the anterior chest wall, may facilitate the procedure and reduce bleeding.

Although many studies show that the complication rate of apex closure are low^[3,10-15]^, it is undeniable that this is one of the main concerns of the Heart Team, especially due to the risk of bleeding. For those experienced in this type of approach, the closure of the left ventricle at the end of the procedure is one of the most delicate parts of the operation. With this in mind, this new device named CorPoint was developed, which aims to minimize the risk of bleeding at the same time as it eases the insertion and manipulation of catheters during surgical procedure.

The device aids in transapical access by presenting the following characteristics:

1. Fast and direct access to the heart and its chambers.
2. Access to the ascending and descending aorta from the apex of the left ventricle.
3. Direct access to the left sided heart valves.
4. Spares the need to suture the heart at the end of the procedure, since a closing capsule is screwed over the base of the device to stop the bleeding.
5. Reduces hemorrhage complications.
6. Reduces operative time.
7. The possibility of enabling this procedure to evolve into a totally percutaneous approach.

## METHODS

### The Device

CorPoint was developed in partnership with HELP Plastic S.A., and is produced entirely of 316 stainless steel, a material largely applied in the medical field since it does not present any adverse reactions to the human body.

**Fig. 1:**
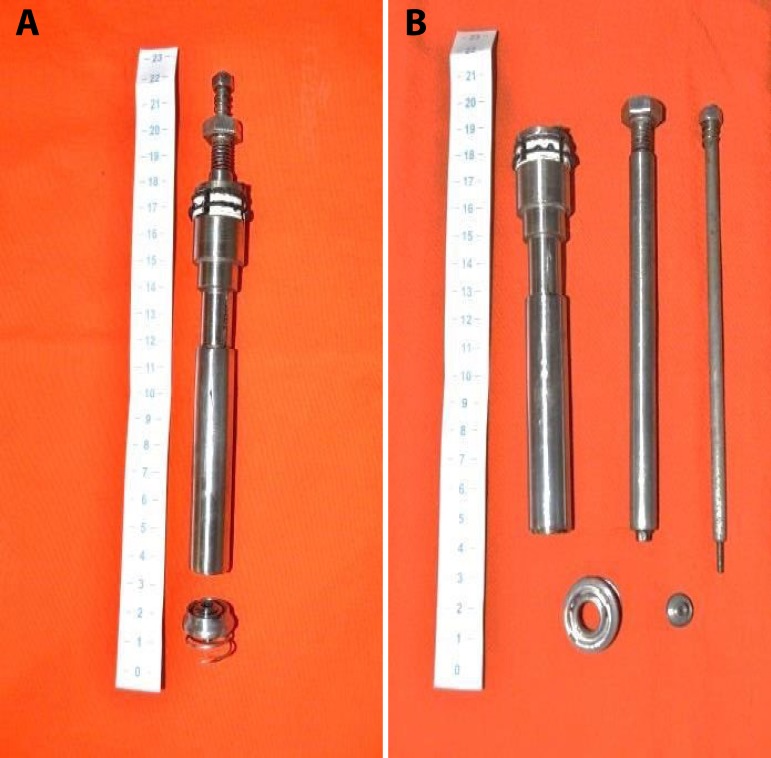
A) CorPoint assembled with the body as one. The base with the coiled spring are apart. B) The device with each of its parts unassembled.

It is composed basically of 3 parts (Figure 1):

1. The body, which represents the introducing system
2. The base, with its coiled spring
3. The closing capsule.

**Fig. 2:**
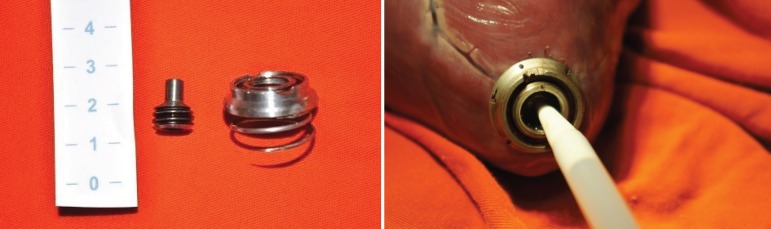
On the left is the base, with its coiled spring and the closing capsule that is screwed over its central opening; and on the right the device is in place, with a valve introducer sheath being passed through it.

The whole device is assembled in one piece. The first step is to puncture the apex of the left ventricle and introduce a guide wire, oriented through fluoroscopy, until it reaches the descending aorta. The wire is then passed inside the CorPoint introducing system and the device is directed towards the heart, where the tip of the spring will touch its surface. The device is then pressed against the beating heart and rotated clockwise, thus allowing the spring to penetrate progressively into the myocardium, until its base reaches the surface (Figure 2). When in position, the base will compress over the myocardium, in a sealed manner, and prevent any type of bleeding.

**Fig. 3:**
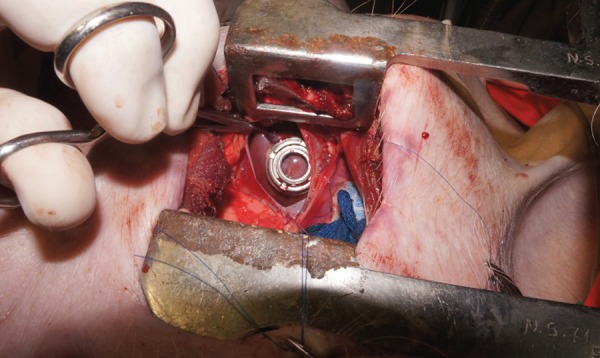
CorPoint implanted on the apex of the left ventricle. Sutures were placed on the pericadium to ease exposure.

The base is circular in shape and has a round opening in its center, through where the catheters are inserted, as well as six small holes in its rim, where sutures can be placed if any bleeding occurs and the surgeon judges that the device needs to be more firmly fixed to the myocardium (Figure 3).

**Fig. 4:**
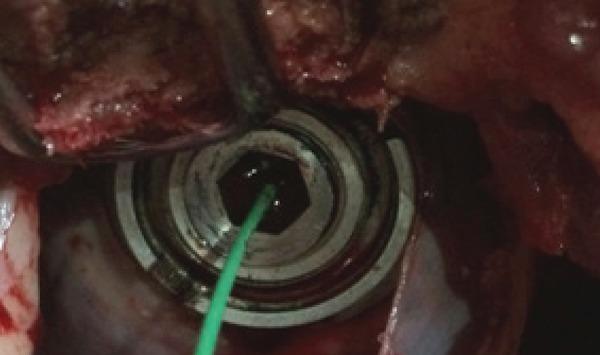
At the end of the procedure, CorPoint is sealed and only the guide wire remains, the last thing to be removed.

The catheters are passed through the body, connected to the base, which provides great stability in regards to their positioning and manipulation. The body possesses a valve mechanism that also prevents bleeding through the interior of the system, especially when the valve insertion catheter is removed and a considerably large perforation is formed in the left ventricle. At this point, at the end of the procedure, the closing capsule is screwed over the central opening of the base and the device is sealed tight (Figure 4). However, the closing capsule presents a small orifice which allows the passage of the guide wire, thus the capsule can be screwed over the base while the guide wire is still in place. This enables the surgeon to remove the guide wire only at the very end of the procedure, which guarantees a much safer and controlled environment.

### Study Design

We developed an experimental study in pigs where the main goal was to validate our device as a viable, safe, fast and effective option for the transapical access of the heart, in the treatment of structural heart diseases. Secondary objectives were to evaluate the bleeding risk, the durability of the device through a new access 30 days after its insertion, and the histopathological alterations in the cardiac muscle related to the presence of the device.

### Specimens

CorPoint was tested in a total of 15 pigs, with a medium weight of 60 kg, nine males and six females. All animals were obtained from a certified farm, properly qualified to provide specimens for scientific research.

Ten animals had the device implanted as an access route for the performance of other endovascular procedures, such as: three TAVI; four procedures on the mitral valve, with the deployment of transcatheter mitral valves and testing of another device for mitral prolapse; and three implants of endovascular aortic endografts on the thoracic descending aorta. All these animals were sacrificed at the end of the procedure.

The other five animals had CorPoint embedded as their main surgical procedure. Catheters and wires were passed through the device to simulate endovascular procedures, but no endovascular prosthesis was deployed. These pigs were kept alive for follow-up with the device fixed to their heart during one month, and at the end of this period were once again operated to test reentry through the same CorPoint that had been inserted previously. Finally, they were sacrificed, their heart explanted and sent to histopathologic analysis.

### Implant of the device

The pigs were submitted to general anesthesia, orotracheal intubation, kept in supine position with their anterior and posterior limbs extended. A small anterolateral thoracotomy approximately 5 cm wide was performed in the fifth intercostal space, dissection performed until the pericardium was reached and incised, to expose the apex of the left ventricle, as seen in Figure 3.

The apex was punctured with a needle at a site not close to any coronary arteries or branches, a hydrophilic guidewire inserted and steered with the aid of fluoroscopy until it reached the descending aorta. Over the wire, a 7 Fr catheter was introduced and the hydrophilic wire was replaced by an extra stiff one, to serve as rail and guide the CorPoint introduction system to the surface of the heart. Progressively thicker catheters were inserted, until the maximum of 24 Fr, to simulate endovascular procedures on the heart valves and aorta, and at the end they were withdrawn and the closing capsule screwed over the base, sealing the device.

### Reentry Assessment

After 30 days, the five animals were taken to the operating room, anesthetized, and placed in the same position. An incision was made on the same spot and CorPoint was easily identified after dissection. The closing capsule was removed and the left ventricle was once again punctured through the central hole on the base, so that catheters were inserted one more time to simulate procedures. The capsule was screwed in place at the end, and, once no bleeding was observed, the specimen was sacrificed and its heart removed for study.

## RESULTS

CorPoint proved to be an excellent tool in the aid of transapical heart access, and proved efficient in both first and redo operations. We were able to perform the proposed operation in all ten animals that had CorPoint implanted as an access route for another main procedure. In these cases, the advantages of the transapical access with minimal blood loss were combined, and in none of the cases the need to place any sutures through the myocardium was observed. Another advantage worth mentioning was the easier manipulation of the heart tip, guaranteed by the body of the device, which allowed for a much more stable handling and easier steering of the wires and sheaths.

With regards to the other five pigs that had CorPoint embedded as their main procedure, none experienced any adverse event during follow-up. All animals returned to the farm where they were kept in specific and controlled conditions, daily monitored and tended to by the team of veterinarians. They all gained normal weight and presented no alterations to their regular behavior.

When submitted to the second surgical procedure one month after the first approach, that is the reentry evaluation, all presented with expected adhesions typical of reoperations. However, these adhesions were restricted to the apex and inferior aspects of the heart. In all pigs, the same previous incision was used and oriented the dissection towards the tip of the left ventricle, with the advantage that the CorPoint was palpable and worked as a landmark to guide the dissection. The closing capsule was easily removed and the left ventricle once again punctured through the central orifice of the device.

Bleeding as a consequence of the insertion of CorPoint was negligible in all 15 cases, and the closing capsule guaranteed hemostasis in 100% of the specimens. The placement of sutures on the myocardium was not necessary in any animal, since CorPoint assured a bloodless field during and after the procedure. Although our device offers six small holes circumferentially on the rim of the device to allow sutures to fix it to the myocardium were proved unnecessary, the coiled spring guaranteed adequate anchoring of the base to the heart surface.

The total amount of blood loss during the procedure was less than 50 ml in 86% of the cases (13/15 specimens). In two animals, a slightly greater amount of bleeding was noted, respectively 150 ml and 220 ml, due to a lesion to the internal thoracic artery during thoracotomy and dissection, which was easily controlled after its ligation.

**Fig. 5:**
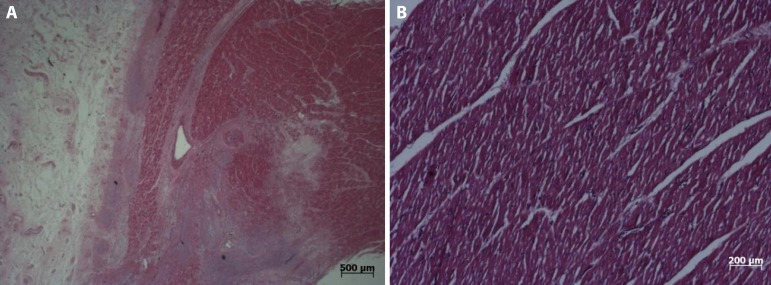
A) Regeneration tissue and fibrosis found in the small area adjacent to the device implant; B) Normal myocardium at the control site.

All five hearts that had CorPoint embedded during one month were sent to histopathological evaluation, after the pigs were euthanized, to assess the structural alterations related to the presence of the device. Two regions were analyzed in each heart: the tip, where the device was implanted, and the basal portion of the inferior wall, far from the implant site, served as control. The area related to CorPoint insertion showed regeneration tissue and fibrosis, both restricted to the areas immediately adjacent to the spring, whereas the control sites showed no alterations, except in one heart that revealed an epicardium thickening (Figure 5). In two cases, an acute inflammatory process related to the CorPoint insertion was found, which may be related to an infection. In one of these cases a small amount of pus was found during tissue dissection. In another heart, the pathologist observed a foreign body reaction with chronic inflammation process. However, all these alterations were limited to areas adjacent to the device, in a way that the muscular tissue between the spirals of the spring was considered normal.

No evidence of thrombus formation on the inner surface of the left ventricle was found, analyzed macro and microscopically. Likewise, no contractile dysfunction related to the presence/ implant of the device was observed, analyzed macroscopically by the surgeon performing the device implant and reentry.

## DISCUSSION

Transapical approach of the heart has been shown as an excellent alternative in minimally invasive procedures and high risk patients, who present with severe cardiovascular diseases, older age and other systemic disorders associated^[16,17]^. In this group, there are patients with heart valve diseases, aortic aneurysms and dissections, congestive heart failure and other infirmities, who will directly benefit from the development of a device that enables such approach in a safer manner, with less risk of complications, bleeding, ventricular rupture and also facilitates posterior interventions. CorPoint was able to match and exceed these expectations in this animal based experimental study, and appears to be a helpful tool for the transapical access of the heart. This becomes even more interesting when we consider its potential application to transcatheter valve-in-valve procedures, frequently performed through transapical access for the treatment of degenerated bioprosthetic heart valves^[18]^.

Regarding this kind of approach, the majority of studies and procedures described in the literature are related to TAVI. However, until this moment there are no available results for a prospective, randomized clinical trial, comparing transapical *versus* transfemoral approach, regarding TAVI or any other endovascular procedure. There are meta-analysis and multicentric studies^[19-22]^ that seem to show a greater 30 day mortality rate related to transapical TAVI (TAAVI) when compared to transfemoral (TFAVI). Nevertheless, these studies also demonstrate that patients submitted to TAAVI carry higher STS and EuroSCORE II risks, which show that they have in fact more comorbidities and higher operative risks. A German study^[23]^ revealed that the STS score was the best long term mortality predictor, regardless of the access route, and that TAAVI, although associated with a higher perioperative mortality, showed no influence on long term prognosis. Other studies also show higher rates of vascular complications related to transfemoral access, alongside with a higher risk of bleeding and blood transfusion^[12,24,25]^.

In any way, a discussion over the best access route for TAVI is not the focus of this study. We wish only to present a tool that aspires to facilitate the transapical access of the heart and, for the time being, is still in an experimental phase of development, but with promising results. Greater studies are required before we can begin to use this technology in human patients.

## CONCLUSION

The transapical approach of the left ventricle and its complications are a worldwide concern. Although there are other devices available on the international market that target this exact problem, such as Apica, Permaseal, EnTourage and CardiApex^[26,27]^; and many of these are already being used in humans with good results, none are available outside Europe.

This is one of the main reasons that led us to develop our own transapical ventricular access and closure device. This CorPoint prototype is the result of a partnership between InCor FMUSP and HELP Plastic Ltda, a Brazilian company, and counted with 100% national technology.

When our national epidemiology is analyzed, cardiovascular diseases appear as our main cause of death and healthcare expenses (DATASUS 2012), aligned with our population aging reality, we can conclude that the cardiovascular burden to society tends only to increase. Therefore, the development of a device that will assist the treatment of such diseases, minimizing risks, complications, and probably ultimately costs, presents as an answer to an ever so growing healthcare demand.

**Table:** 

Authors' roles & responsibilities	
LP	Substantial contributions to the conception or design of the work; or the acquisition, analysis, or interpretation of data for the work; agreement to be accountable for all aspects of the work in ensuring that questions related to the accuracy or integrity of any part of the work are appropriately investigated and resolved; final approval of the version to be published
JHPF	Substantial contributions to the conception or design of the work; or the acquisition, analysis, or interpretation of data for the work; drafting the work or revising it critically for important intellectual content; final approval of the version to be published
FVAJ	Substantial contributions to the conception or design of the work; or the acquisition, or interpretation of data for the work; final approval of the version to be published
PSG	Substantial contributions to the conception or design of the work; or the acquisition, analysis, drafting the work or revising it critically for important intellectual content; final approval of the version to be published
LFPM	Drafting the work or revising it critically for important intellectual content; final approval of the version to be published
FBJ	Final approval of the version to be published
